# Intra- and inter-multi-omics interaction analysis using deep learning

**DOI:** 10.1093/bioadv/vbag165

**Published:** 2026-07-09

**Authors:** Mingon Kang, Eunyoung Jang, Tesfaye B Mersha

**Affiliations:** Department of Computer Science, University of Nevada, Las Vegas, NV 89154, USA; Department of Computer Science, University of Nevada, Las Vegas, NV 89154, USA; Precision Pulmonary Medicine Research Program, Department of Medicine, Division of Pulmonary, Critical Care, and Sleep Medicine, Indiana University School of Medicine, Indianapolis, IN 46202, USA

## Abstract

Multi-omics interactions, including intra- and inter-omics interactions as well as socio-environmental influences, are key to uncovering molecular mechanisms that may be missed by individual omics analysis or conventional integration approaches. Deep learning offers a promising solution to overcome current limitations, including the complexity of modeling high-dimensional, nonlinear interactions, limited sample size and multiple testing burden.

Recent advances in high-throughput multi-omics analysis enhance the ability to represent quantitative characteristics of various biological processes in transcriptomics, microbiomes, proteomics, and metabolomics. This results in comprehensive data-driven analysis and increased predictive power beyond individual omics. Deep Learning (DL) algorithms have recently provided efficient solutions for multi-omics analysis by dynamically learning features from raw data using multilayer hierarchical structures (Kang *et al.* 2022). DL models capture complex and nonlinear hierarchical feature representations that are often missed by existing benchmark models. Naïve integrative approaches analyze multi-omics data individually and then integrate complementary information from multiple data sources. However, these studies perform integration analysis under the assumption that each dataset has independent biological mechanisms. Consequently, they often ignore multi-omics interactions that represent relationships among different datasets. Furthermore, most DL-based multi-omics data analyses focus on representing the group effects of the same omics using dimensionality reduction techniques (e.g. PCA or autoencoder) and combining high-level embeddings of each omics analysis. This approach leads to a critical lack of model interpretability in multi-omics data analysis. Currently representative deep learning-based multi-omics interaction models are summarized in Table 1.

**Table 1:** Deep learning-based multi-omics interaction models.

Types	Core module	Models	Omics types	Strength	Weakness	Ref
Pathway-based inter-omics interaction	Sparse NN	MiNet	RNA-seq, CNV, DNAm	Explicit modeling for inter-omics interaction for interpretation	High computation	Hao *et al.* 2019
	Sparse NN	DeepOmix	mRNA, DNAm, CNV, Mutation	Explicit modeling for inter-omics interaction for interpretation	Limited modeling of complex cross-omics interactions	Zhao *et al.* 2021
	CNN	PathCNN	mRNA, CNV, DNAm	Interpretable multi-omics integration using pathway images	Performance sensitive to pathway-to-image encoding design	Oh *et al.* 2021
	Attention-based sparse NN	DeepKEGG	mRNA, miRNA, SNV	Interpretable pathway-guided multi-omics integration	Feature-level fusion limits interaction modeling	Lan *et al.* 2024
Variable/embedding-based integration	Hybrid CNN + sparse NN	PAGE-Net	lncRNA, WSI	Integration for structured and unstructured datatypes	Intra-gene interactions considered; inter-modal interactions ignored	Hao *et al.* 2020
	UMAP-based NN with density-based clustering	GAUDI	mRNA, miRNA, DNAm /scRNA-seq, scATAC-seq	Interpretable latent embedding for multi-omics clustering	Limited end-to-end supervised learning due to embedding + cluster pipeline	Castellano-Escuder *et al.* 2025
Pathway-hierarchy interactions	Multi-layered hierarchical network	P-Net	CNV, Mutation	Hierarchical pathway-informed interpretability	Limited to sparse omics data	Elmarakeby *et al.* 2021
		MULGONET	mRNA, DNAm, CNV		High model complexity	Lan *et al.* 2026
		IBPGNET	CNV, Mutation		Limited to sparse omics data	Xu *et al.* 2024
Graph-based interactions	Multi-modal gene-gene interactions (GCN)	multilevel GNN	mRNA, DNAm, CNV	Multi-omics interaction graph supports gene/pathway-level interpretation	Dependent on mutual-information gene selection	Yan *et al.* 2024
	Pathway-pathway interactions (GCN)	Cox-Path	mRNA, DNAm	Pathway-level interactions for prognostic interpretability	Limited to similar omics types	Ma *et al.* 2024
		GNNRAI	RNA-seq, Protein abundance data	PPI-guided representation enhances biological interpretability	Dependent on PPI networks	Tripathy *et al.* 2025
	Pathway-pathway interactions (GAT)	GraphPath	CNA, Mutation	Interpretable pathway interaction modeling	Limited to sparse omics data	Ma and Wang 2024
Feature-selection-based interactions	Associative GAT	AMOGEL	mRNA, miRNA, DNAm	Rule-based feature reduction with adaptive graph construction	Potential information loss due to rule-based feature reduction	Tan *et al.* 2025

** DNAm, DNA Methylation; mRNA, mRNA expression; miRNA, miRNA expression; lncRNA, long non-coding RNA expression; WSI, histopathological WSI.

Multi-omics interaction methods that combine information from diverse omics and non-omics data sources facilitate the assessment of information flow from one omics layer to another. Further, they elucidate the intricate interplay between various molecular profiles to address disease complexity. However, current multi-omics approaches seldom considered complex interaction effects, which hinders their applicability in medicine and public health. Without such methods, the promise of translating interaction study results will likely remain unfulfilled. Although explicit modeling of cis- (or trans-) acting regulation and attention-based interaction representations are examples of DL-driven identification of multi-omics interactions, the main challenges of complex interaction analyses include computational complexity, limited sample size, and nonlinearity of interactions. For instance, conventional genome-wide analyses scan pairwise main effect interactions in the entire genome. However, most studies to date are underpowered due to small sample sizes and the burden of multiple testing. Furthermore, models or strategies to detect nonlinear interactions are lacking.

Gene–environment interactions have traditionally been defined as the interplay between genetic variation and environmental exposures in influencing disease risk. However, in the multi-omics era, they are redefined as complex, dynamic, context-defining biological inputs across molecular layers in which genetic, epigenomic, transcriptomic, and environmental factors jointly interact to modulate gene regulation, cellular states, and downstream biological pathways. This approach enables the identification of context-dependent interactions across molecular layers and improves the mechanistic interpretation of disease processes. Hence, simple combinatorial effects of features by DL do not necessarily represent biological interactions. Addressing these challenges requires robust embedding strategies, temporal modeling approaches, and integrative methods that enable coherent and biologically meaningful cross-modal learning. Furthermore, it should be noted that integrating environmental exposures with other omics data introduces specific technical challenges, including the mismatch between low-dimensional exposure data and high-dimensional often continuous omics data, as well as the temporal variability of exposures, noise, and missingness.

In this article, we propose multi-omics approaches to explore inter- and intra-omics interaction effects along with socio-environmental risk factors (Fig. 1). In multi-omics research, inter-omics and intra-omics interactions refer to different types of biological relationships across or within layers of omics data. Inter-omics interactions refer to relationships between different layers of omics data, such as how genetic variants (genomics) interact with gene expression (transcriptomics), how mRNA levels interact with protein abundance (proteomics), or how enzyme levels interact with metabolite concentrations (metabolomics). These interactions help uncover cross-level regulatory mechanisms, such as epigenetic modifications affecting transcription. In contrast, intra-omics interactions occur within the same omics layer, such as gene co-expression networks in transcriptomics, protein-protein interactions in proteomics, or metabolite correlations within metabolic pathways in metabolomics. Both types of interactions are crucial for understanding complex biological systems and disease mechanisms.

**Figure 1:**
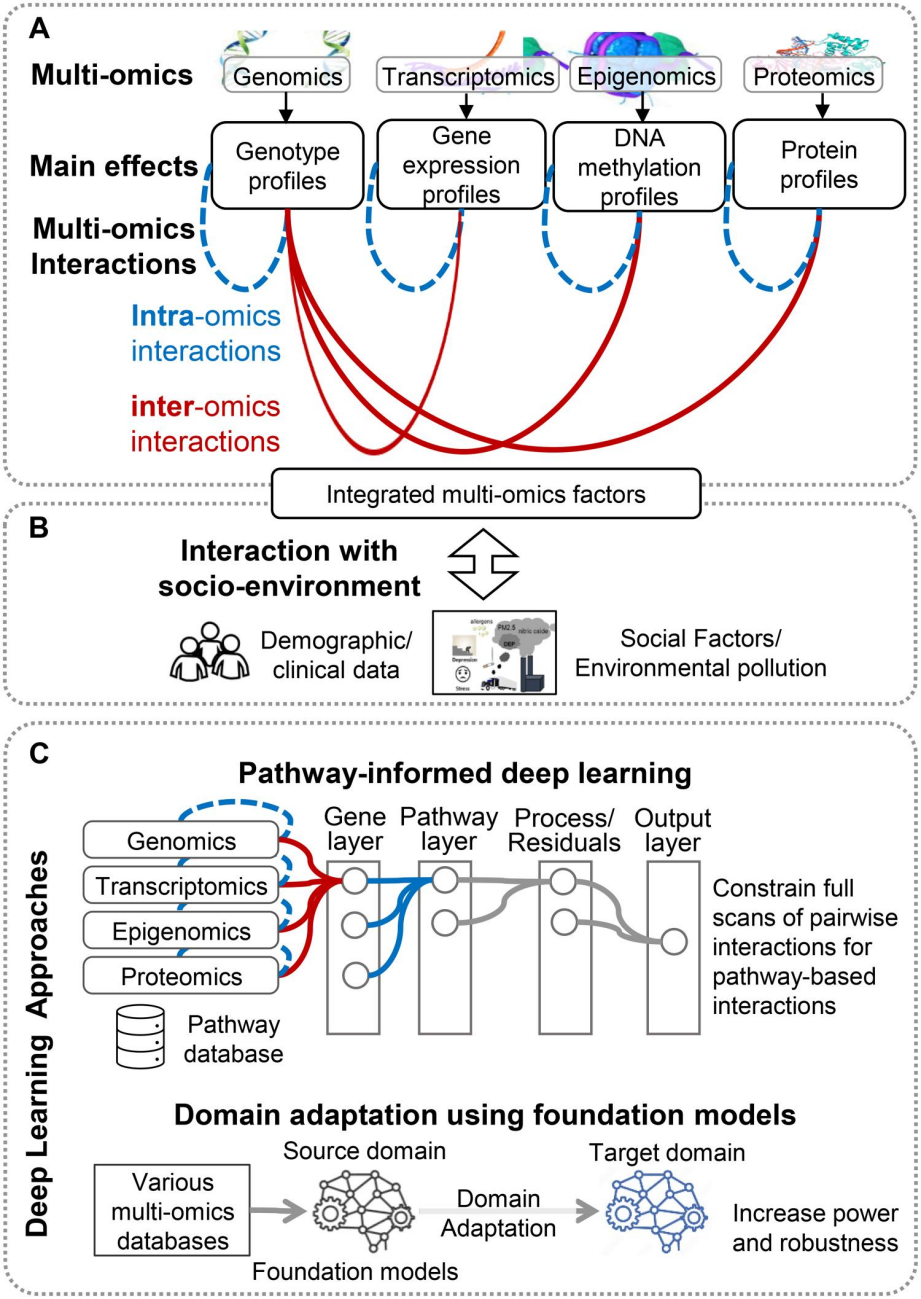
Multi-omics data represents quantitative characteristics of various biological processes in genomics, transcriptomics, epigenomics, and proteomics. Potential solutions to increase power and robustness by analyzing intra- and inter-omics interactions (A) and interactions with socio-environmental risk factors (B) include leveraging pathway-informed deep learning and domain adaptation using foundation models (C).

There are two potential approaches to harness inter- and intra-omics interactions: (1) knowledge-informed modeling and (2) domain adaptation strategies using foundation models (Fig. 1C). First, knowledge-informed modeling utilizes prior biological knowledge to build interpretable model architecture or profile potential interactions of interest. For instance, pathway-informed DL (PIDL) models incorporate well-curated biological pathway knowledge (e.g. KEGG, Reactome databases) into neural network architectures for explicit and intuitive biological interpretability (Selby *et al.* 2025). PIDL involves pathway layers, where each node explicitly represents corresponding biological pathway enrichment with sparse connections based on the established gene and pathway relationships (Fig. 1C). The explicit sparse connections between the gene and pathway layers constrain the scans of pairwise interactions, focusing on interactions of pathway-related genes, since examining all pairwise interactions is computationally infeasible. The pathway layers are structured in various manners, as simple pathway-layer (PASNet (Hao *et al.* 2018), MiNet (Hao *et al.* 2019), PAGE-Net (Hao *et al.* 2020), PINNet (Kim and Lee 2023), PathCNN (Oh *et al.* 2021), DeepKEGG (Lan *et al.* 2024), DeepOmix (Zhao *et al.* 2021)), pathway-hierarchy (P-Net (Elmarakeby *et al.* 2021), MULGONET (Lan *et al.* 2026), IBPGNET (Xu *et al.* 2024)), and pathway-pathway graph (Multilevel GNN (Yan *et al.* 2024), GraphPath (Ma and Wang 2024)). Moreover, the inter- and intra-omics interactions can be modeled by the sparse connections within the different layers.

Specifically, MiNet’s architecture focuses on examining of cis-acting regularization for inter-omics interaction in the multi-omics layer (e.g. SNPs of upstream or downstream within 20 Kb interacting with a gene), while PASNet and P-Net constrain intra-omics interactions between genes in the same pathway. PathCNN analyzes inter-omics interactions from pathway images using convolutional neural networks (CNN). This approach focuses on biologically meaningful interactions rather than full pairwise scans. Moreover, these PIDL models can leverage the inherent advantages of DL that effectively capture nonlinear interaction effects of genes and pathways. However, the pathway-based DL has limitations, such as incomplete pathway knowledge and tissue/population heterogeneity. Although pathway databases are becoming more comprehensive and growing, they remain limited by a reliance on existing knowledge, as unannotated genes are discarded and analysis becomes inherently biased.

Second, domain adaptation leveraging large foundation models (FM) is another potential strategy to address the lack of power due to limited sample sizes, enabling effective learning of complex interactions. Domain adaptation (DA) is inspired by the human cognitive process of generalizing new concepts with scarce data samples in a domain (i.e. target domain) based on rich datasets in a related domain (i.e. source domain). Foundation models, trained with publicly available large multi-omics databases (e.g. TOPMed, All of Us Research Program, and TCGA), can serve as a starting point to infer generalized biological models, which can then be applied to various downstream tasks in the target domain (Cui *et al.* 2024; Waqas *et al.* 2024; Yuan *et al.* 2025). This approach can efficiently optimize DL models that require large parameters for interactions, ultimately increasing the power to detect interactions with modest sample sizes. Furthermore, DL models, trained by domain adaptation with large foundation models, would be beneficial to avoid model biases to the limited target data and to provide reproducible performance on related new datasets. However, the primary challenges of the domain adaptation strategy arise from domain shift between source and target populations (e.g. platform differences and ancestry composition), necessitating more robust solutions. Furthermore, current foundation models are based on generative AI techniques and zero-shot evaluation revealed its limitations (Kedzierska *et al.* 2025). Rigorous evaluation and their reliability are required to deploy foundation models to downstream tasks. The launching of large-scale biobanks and observational studies, such as the All of Us Research Program, and other initiatives are helping address sample size limitations by providing extensive genetic and environmental data across diverse populations.

Building reliable foundation models requires training with large, high-quality multi-omics datasets and solutions for robust data harmonization and heterogeneous data integration. First, high-quality multi-omics data directly determines the quality and potential implications of foundation models. Such data should be biologically consistent and clinically relevant. Quality control for each omics data type must meet its own field-specific standards. Multi-omics analysis relies on the assumption that the different data layers originate from the same biological source. The availability of clinical metadata (e.g. demographic features) is critical for fairness analysis. Second, robust data harmonization allows for combining multiple datasets to learn generalized interaction patterns while removing batch effects. Heterogeneous data integration also requires solutions for platform-driven missing value problems, scaling mismatches, and interpretability. More importantly, foundation models require explicit modeling of inter- and intra-omics interactions. Most current models focus on the main effects of each omics factor or the group effects of intra-omics interactions independently. Biologically interpretable multi-omics interaction modeling in foundation models directly impacts downstream tasks through domain adaptation.

While multi-omics information plays a crucial role in shaping an individual’s risk for these diseases, environmental influences, such as diet, pollution, stress, and lifestyle choices, can modify the way omics function, influencing disease onset and progression. For example, Asthma is strongly influenced by gene-environmental interactions. Individuals with the GSTM1 null genotype show increased asthma risk, when exposed to air pollutants like diesel exhaust (Dai *et al.* 2021). The CD14 TT genotype is linked to reduced atopic asthma risk in children exposed to endotoxins, supporting the hygiene hypothesis (Martinez 2007). ADAM33 variants heighten asthma susceptibility in children exposed to tobacco smoke (Reijmerink *et al.* 2009), while IL4RA variants may protect farm-raised children due to early microbial exposure (Johansson *et al.* 2019), ORMDL3 variants at 17q21 are associated with childhood asthma, especially following early viral infections (Ono *et al.* 2014). These examples highlight how genetic predispositions can amplify or buffer environmental effects, informing precision medicine—similar to how BRCA1/2 mutations guide PARP inhibitor use and HER2 expression informs trastuzumab therapy (Olopade *et al.* 2008).

Deciphering the interplay between multi-omics data and socio-environmental exposures is essential for uncovering the underlying biological mechanisms and enhancing the translational relevance of research in complex diseases such as asthma (Wu *et al.* 2023). There are numerous statistical and machine learning approaches, such as individual-level polygenic scoring, latent variable models, and network-based approaches, to assess gene-environment interactions in human studies (Herrera-Luis *et al.* 2024). Identification of socio-environmental exposures to prioritize functionally relevant risk factors and biological pathways could enhance G × E interaction analyses by increasing statistical power and mitigating the multiple testing burden. DL-powered multi-omics-environmental exposure interaction algorithms simultaneously estimate the main effects and G × E interactions by overcoming hierarchical constraints in conventional regression-based models and can process complex, high-dimensional omics data, identifying hidden patterns that would be impossible to detect using traditional statistical approaches. There are also several challenges that must be addressed including measurement variability, co-linearity among omics and efficient methods to integrate and conduct interaction with non-omics datasets.

In summary, despite advances in using AI in multi-omics research, current approaches primarily integrate complementary main effects of multi-omics data. In this commentary, we show that multi-omics interactions (i.e. intra-omics and inter-omics interactions) are critical, but limited due to sample size, multiple testing, and complex relationships to model interactions. To mitigate these challenges, we suggest potential DL-based solutions, such as pathway-informed analysis and domain adaptation using foundation models. However, these AI-driven interaction analysis models need to be rigorously tested to avoid bias in training datasets, lack of transparency in decision-making processes, and privacy concerns, to ensure fairness, ethics, and applicability across diverse populations. Furthermore, key challenges include developing cross-population transfer learning frameworks to ensure interaction models are generalizable across diverse cohorts and environments. Integrating interaction models and causal inference with deep learning should help us move beyond simple correlations and toward identifying true mechanistic drivers of disease. This requires overcoming the inherent domain shifts between populations while maintaining the computational rigor needed to distinguish causal regulatory links from high-dimensional noise. By developing equitable and interpretable AI multi-omics interaction models, researchers can harness the power of DL to make precision medicine a reality for all.

## Author’s contributions

MK and TM conceptualized this study and mainly wrote the original and revised manuscript together. MK visualized the concept. EJ surveyed related works and investigated literature and data. TM supervised this study and acquired funding. All authors read and approved the final manuscript.
